# A Study of the Relationship between Polymer Solution Entanglement and Electrospun PCL Fiber Mechanics

**DOI:** 10.3390/polym15234555

**Published:** 2023-11-28

**Authors:** Manasa Rajeev, Christine C. Helms

**Affiliations:** Department of Physics, University of Richmond, Richmond, VA 23173, USA

**Keywords:** electrospinning, lateral AFM, strength, polycaprolactone, entanglement

## Abstract

Electrospun fibers range in size from nanometers to micrometers and have a multitude of potential applications that depend upon their morphology and mechanics. In this paper, we investigate the effect of polymer solution entanglement on the mechanical properties of individual electrospun polycaprolactone (PCL) fibers. Multiple concentrations of PCL, a biocompatible polymer, were dissolved in a minimum toxicity solvent composed of acetic acid and formic acid. The number of entanglements per polymer (*n_e_*) in solution was calculated using the polymer volume fraction, and the resultant electrospun fiber morphology and mechanics were measured. Consistent electrospinning of smooth fibers was achieved for solutions with *n_e_* ranging from 3.8 to 4.9, and the corresponding concentration of 13 g/dL to 17 g/dL PCL. The initial modulus of the resultant fibers did not depend upon polymer entanglement. However, the examination of fiber mechanics at higher strains, performed via lateral force atomic force microscopy (AFM), revealed differences among the fibers formed at various concentrations. Average fiber extensibility increased by 35% as the polymer entanglement number increased from a 3.8 *n_e_* solution to a 4.9 *n_e_* solution. All PCL fibers displayed strain-hardening behavior. On average, the stress increased with strain to the second power. Therefore, the larger extensibilities at higher *n_e_* also led to a more than double increase in fiber strength. Our results support the role of polymer entanglement in the mechanical properties of electrospun fiber at large strains.

## 1. Introduction

Electrospun nanofibers hold the potential for applications in many fields, including textiles, filtration, photonics, electronics, medicine, material engineering, and tissue engineering [1,2,3,4,5,6]. However, many of these applications require specific fiber mechanical properties. For example, when using electrospun fibers in tissue engineering of cell scaffolds, the mechanical properties of the fibrous scaffold affect cell survival, proliferation, and migration and, therefore, must be tailored to match the application [7,8,9]. Additionally, the mechanical properties of electrospun fibers are important in their applications as reinforcements in composite materials [10,11]. More generally, electrospun fibers must be able to withstand the forces and pressures exerted by their environment during their lifespan as a textile, filter, scaffold, etc. Therefore, measuring, controlling, and understanding the mechanisms related to the mechanical properties of electrospun fibers and mats is important in realizing their potential.

A body of research has been built by numerous labs working to gain knowledge necessary for tailoring the mechanical properties of individual electrospun fibers and full mats [12,13,14,15,16]. Research into individual fiber mechanics has shown that the modulus of electrospun fibers is dependent on polymer type, polymer molecular weight, polymer concentration, and fiber diameter [17,18,19,20,21].

The production of electrospun fibers is sensitive to multiple solution parameters, such as the viscosity, molecular cohesion, and dielectric properties of the initial polymer solution [22,23,24,25]. The flow behavior and viscosity of polymeric solutions and melts are directly related to polymer entanglement [26]. The number of entanglements per polymer, *n_e_*, can be altered by changing the solvent, the molecular weight of the polymer, and the concentration of the polymer.

The interactions between polymer and solvent are complex and can lead to substantial changes in polymer conformation in solution [27]. As polymer molecular weight and concentration increase, interactions between the polymer and solvent system begin to play a more substantial role in the rheological properties of the solution [28]. These complex polymer-solvent interactions, along with the molecular weight of the polymer, determine the polymer radius. As a polymer solution’s concentration increases from dilute to semi-dilute, polymers begin to overlap, and the system can be represented as a series of correlated blobs, known as the blob model [29]. The blob model, using elements of both a random walk and a self-avoiding random walk, results in a critical entanglement molecular weight and entanglement density that depend on polymer concentration, solvent interactions, and molecular weight [30,31].

When a polymer is diluted by the addition of a solvent, the entanglement molecular weight $M_{e}$ (the average molecular mass between two entanglements) increases. The entanglement molecular weight in solution can be calculated using the diluted polymer volume fraction and the entanglement molecular weight of the melt [31,32,33,34].

(1) $${\left(M_{e}\right)}_{soln}=\frac{{\left(M_{e}\right)}_{melt}}{{\phi_{p}}^{\alpha }}$$

(2) $$\phi_{p}=\frac{V_{p}}{V_{tot}}=\frac{c}{\rho_{p}},$$

where ${\left(M_{e}\right)}_{melt}$ is the entanglement molecular weight of the PCL melt, 2500 g/mol [35], $\phi_{p}$ is the diluted polymer volume fraction, and α is the dilution exponent. $\phi_{p}$ is the ratio of the volume of the polymer to the total volume of the mixture and is dependent on the concentration of the polymer, *c*, and its density, $\rho_{p}.$ The dilution exponent represents changes in polymer swelling due to the solution and its motion due to interactions with neighboring polymer chains [36]. We assume a dilution exponent of 1, although its value is reported between 1 and 2.25 [30,31,32,37,38].

Once the entanglement molecular weight is known, we can determine the number of entanglements per polymer in solution by dividing the molecular weight by the weight per entanglement,

(3) $$n_{e}=\frac{M_{W}}{{\left(M_{e}\right)}_{soln}}$$

where $M_{W}$ is the molecular weight of a polymer chain.

Because of the relationship between entanglement, viscosity, and fiber production, research groups have studied the role of entanglement in the production of electrospun fibers [39,40,41]. Shenoy et al. showed polymer entanglement was required to produce electrospun fibers from polymers in good solvent with non-specific polymer–polymer interactions. Further, they showed that for many polymer–solvent combinations, a minimum of approximately 3.5 entanglements per molecule was needed to produce smooth fibers without beading [41].

During electrospinning, the polymer solution undergoes rapid axial stretching due to ejection through the needle, the pull of the electric field, and subsequent whipping due to Coulombic instabilities. The Plateau–Rayleigh instability causes radial contraction, which is further promoted by rapid solvent evaporation that leads to retention of the final polymer solution configuration in the solid fibers deposited on the collector [42]. While the electrospinning process likely induces elongation and some disentanglement of the polymer strands, the final fiber properties are intimately related to the number of entanglements in the initial solution.

Studies have investigated the effect of polymer concentration on the mechanical properties of electrospun fibers. However, the results often focus on the strong dependence of fiber modulus and strength on fiber diameter [20,43,44,45,46]. A recent study by Peng and Mirzaeifar used molecular dynamics simulations to compare fibers formed from differing molecular weight polymers at similar concentrations and fiber diameters, thereby differing the number of entanglements while holding other parameters constant. This study predicted an increase in modulus and strength of fibers formed with higher molecular weights at constant polymer densities [47].

To the best of our knowledge, there are limited experimental studies focusing on and showing the effect of polymer solution entanglement or concentration on the mechanical properties of electrospun fibers of the same diameter. A study by Lim et al. briefly reported differences in the stress-strain behavior of fibers with similar diameters formed with various concentrations of PCL and attributed these differences to changes in the crystalline structure in the fiber [20]. However, the role of the crystal structure is inconclusive, as the measurement of crystallinity and mechanics was made on different groups of fibers.

In this paper, we investigate the role of polymer solution entanglement number on the mechanics of electrospun polycaprolactone (PCL) nanofibers. We selected PCL for its biodegradability and biocompatibility and a less toxic solvent, 3:1 acetic acid to formic acid. We found that polymer entanglement alters electrospun fiber mechanics especially at high strains.

## 2. Materials and Methods

### 2.1. Polymer Solution Preparation and Viscosity

Polycaprolactone pellets (M_n_ 80,000, PDI < 2, Sigma-Aldrich, St. Louis, MO, USA) were dissolved in a 3:1 *v*:*v* solution of acetic acid (ACS reagent grade) and formic acid (88%). To allow for full dissolution, especially at high concentrations, the polymer solution was stirred for three hours before use. The solution’s dynamic viscosity was measured using a Vevor NDJ-9S rotational viscometer (Vevor, Rancho Cucamonga, CA, USA).

### 2.2. Electrospinning

For electrospinning, a 6 mL syringe was used to pump the PCL solution into a one inch (27 gauge, I.D. 0.21 mm) blunt needle. A syringe pump (New Era Model NE-300, Farmingdale, NY, USA) kept the pump rate at a constant speed of 1 mL/hour for each electrospun sample. A voltage of 12 kV was applied to the needle (Gamma High Voltage) and a collector distance of 12.5 cm was used between the needle and the grounded sample collector. The humidity and temperature of the spinning environment were monitored (Ets Dual Control Model 5200, Electro-Tech Systems Inc, Perkasie, PA, USA). The humidity remained at 53 ± 1% and the temperature was 22 ± 1 °C. Fibers were spun and collected onto glass slides (CS-8R cover glass) for SEM (scanning electron microscopy) analysis or onto pre-made striated substrates on thin glass slides for the manipulations of the fibers with AFM. The glass slides and striated substrates were place immediately on top of the larger grounded collector.

### 2.3. Diameter Measurements by SEM

Samples for SEM use were collected on small glass slides placed atop the grounded collector. The slides were mounted with carbon tape onto cylinders and sputter-coated with gold palladium. Images were acquired using a JEOL 6360 LV SEM (JOEL USA, Inc, Peabody, MA, USA) at magnifications ranging from 6000 to 10,000 and with a voltage of 15 kV.

Fiber diameters were measured using Image J (version 1.54d). Samples were collected at low density, so that each image only had a few fibers, and all the fibers in an image were measured. We used the Kolmogorov–Smirnov test to test the diameter data for normality.

### 2.4. AFM Substrate Preparation

Sample substrates were prepared using a micromoulding technique, as previously described [48]. Briefly, a silicon wafer was etched with the desired substrate pattern. A negative of this wafer was produced and used as a stamp for substrate preparation. Stamps were created using a 10:1 SYLGARD 184 Silicone Elastomer Base monomer and a SYLGARD 184 Silicone Elastomer Curing Agent catalyst which were mixed and poured over the etched silicon wafer in a weighing boat. A vacuum was used to remove any bubbles present on the surface. The SYLGARD polymer was allowed to cure for 30–60 min in the oven at 70 °C until the polymer hardened. The silicon wafer was then gently peeled off, and the desired stamps were cut out and stored in 2% sodium dodecyl sulfate.

Small drops of UV-curing glue (Norland optical adhesive 81) were placed on top of glass cover slides (No. 1.5), and the stamps prepared as described above were pushed into the glue. The cover glass with stamp and glue was placed on a UV light source until cured, and the stamp was removed leaving behind a striated substrate with a repeating pattern of 10 µm wide ridges and 12 µm wide and approximately 7 µm deep wells.

### 2.5. AFM Measurements—Three-Point Bending

**Figure 1:**
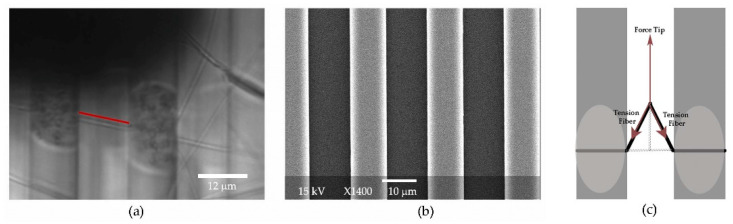
(**a**) Optical image of electrospun fibers on the striated surface. The dark shadow at the top is created by the cantilever. The vertical stripes are the ridges of the striated surface, where discoloration is seen near the fiber of interest due to the application of glue to the ridges. The glue was only applied for lateral force measurements. The ridges provide a calibration standard for the image. The red line indicates the length of the fiber, in this case 12.6 microns. (**b**) An SEM image of the striated surface, where the gap between the ridges is consistently measured to be 12 μm. (**c**) Schematic of the geometry of lateral fiber manipulation. The fiber shown in black is anchored to the gray ridges with glue.

To measure Young’s modulus, a three-point bending technique was utilized by pushing the center of the fiber between two ridges using an AFM cantilever (NSC35/AlBS tip C). The length of the fiber between the ridges and the center of the fiber was determined by optical microscopy imaging combined with known dimensions of the substrate and precision positioning using AFM software (Asylum MFP3D 14.20.152 and inhouse controlled step script available at: https://facultystaff.richmond.edu/~chelms/publications.html, accessed on 5 October 2023), see Figure 1. Briefly, the location of the edge of the ridge was determined using the force response of the AFM cantilever in contact with the surface. The tip was then moved to the center of the well, considering the geometry of the substrate and fiber system, and using the AFM piezo closed-loop positioning. The tip location was further confirmed via optical imaging.

The fiber diameter was determined by imaging the fiber with the AFM in tapping mode on the ridge adjacent to the manipulation. The AFM tip was calibrated for force measurements using the built-in calibration system, which is based on cantilever deflection from a hard surface, and a thermal calibration method. We fit the data to three-point bending theory for unfixed ends (Equation (1)) to determine the young’s modulus, *E*, of each fiber.

(4) $$E=\frac{L^{3}}{48I}\frac{dF}{dz},$$

where *L* is the length of the fiber, *I* is the moment of inertia for a circular cross section, and *dF*/*dz* is the force versus deflection curve, with the distance travelled in *z* corrected for deflection of the cantilever.

### 2.6. AFM Measurements—Lateral Manipulation

To probe fibers at a higher strain using lateral force AFM, the fibers were glued to the ridges using a “dip-pen” method and Loctite Marine Epoxy glue [49]. The epoxy was mixed with a higher ratio of hardener to achieve a viscosity that was ideal for transfer. To precisely add drops of glue to the fibers, a blunt AFM tip with a single cantilever was used to pick up the glue and place it on the ridges to secure the fiber. The epoxy was allowed to set overnight before proceeding with fiber manipulations.

An AFM cantilever (NSC35/AlBS tip C, NanoandMore, Watsonville, CA, USA) was calibrated for lateral force,

(5) $$F_{lat}=k_{lat}∆x$$

where $k_{lat}$ is the spring constant of the cantilever and ∆x is the displacement of the cantilever tip. Briefly, a lateral voltage versus tip displacement curve was obtained by pushing the tip against a glass ridge. Then Euler–Lagrange beam mechanics with a correction for the z location of the applied force and length of the cantilever to tip were used to calculate the torsion constant by

(6) $$k_{lat}=\frac{Gwt^{3}}{{3L(h+\frac{t}{2}-d)}^{2}}\left(\frac{L}{L\prime }\right)$$

where *G* is the shear modulus of the cantilever, *w* is the width, *L* is the length, *L*′ is the length to the tip, *t* is the thickness, *h* is height of the tip, and *d* is the depth of the cantilever in the *z* direction (often 2 μm). The length, width, and height are found using the optical microscopy images of the cantilever. The thickness is found using the relationship between the normal spring constant, *k_n_*, and thickness, *t*, given by Euler–Bernoulli beam mechanics.

(7) $$k_{n}=\frac{Ewt^{3}}{4L^{3}}$$

The normal spring constant is found using AFM software (Asylum MFP3D 14.20.152), as described above.

The force applied to the fiber can be determined and used to find the stress.

(8) $$\sigma =\frac{F_{lat}}{A}=\frac{F_{lat}}{\pi r^{2}}$$

where *A* is the cross-sectional area of the fiber, and *r* is the radius of the fiber, determined by imaging the fiber with the AFM in tapping mode on the ridge adjacent to the ridge where the fiber was glued.

The cantilever tip was positioned next to the fiber in the center of the well. The movement of the cantilever was controlled using a homemade script named Controlled Step, which allowed the cantilever to be maneuvered in steps of selected size and stepping rate. The strain rate for fiber manipulation was held constant at 200 nm/s. The fibers glued at the ridges were pulled laterally until they ruptured. A schematic of the lateral force manipulations can be found in the work by Baker et al. [50].

### 2.7. Statistical Analysis

Lateral force measurements were used to calculate maximum strain, stress, and initial modulus for each fiber. Calculations were performed in Microsoft Excel and Mathematica, and a two-tailed *t*-test was used to determine significant differences between varying populations of PCL. The mechanical property measurements had large variability. However, some data were identified as outliers. Data points that were two or more standard deviations from the calculated average were considered outliers and were not included in the analysis. The extensibility and maximum stress of the 13 g/dL PCL and 17 g/dL PCL had one outlier each.

## 3. Results

### 3.1. Degradation of the Polymer in Acetic: Formic Acid (3:1)

**Figure 2:**
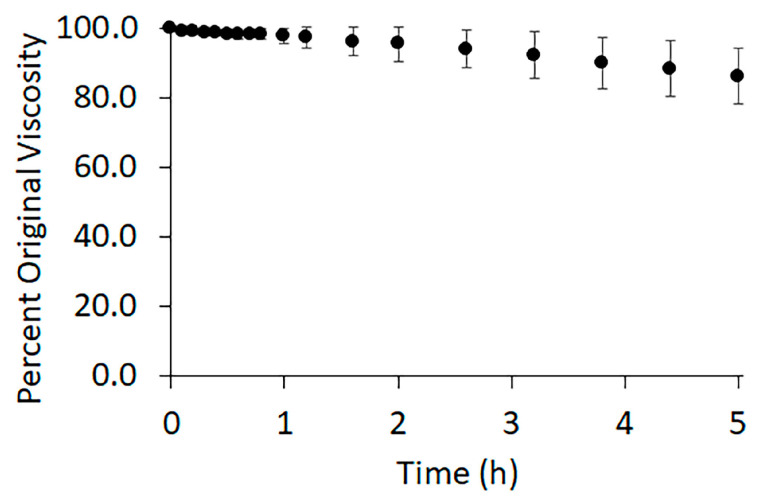
Average percent viscosity of 10 g/dL PCL in 3:1 acetic acid: formic acid. The data is an average over three trials with error bars representing the standard deviation. The zero-time point indicates the moment the solution completed its 3 h of mixing. The data was recorded for five hours.

Polymer degradation can greatly reduce polymer entanglement. The low toxicity advantage of acetic acid/formic acid solvent systems is somewhat diminished due to their known hydrolytic degradation of PCL [51,52]. To monitor polymer degradation, we measured solution viscosity over time (Figure 2). After three hours of stirring, the time allowed for the polymer to fully dissolve, the viscosity of the solution was continuously recorded for 5 h. A slow decrease in viscosity was observed over time, indicative of degradation. As the polymer degraded, the variance between the samples increased. The standard deviation of the measured viscosity remained within the uncertainty of the viscometer for the first hour following dissolution. After two and a half hours, the average viscosity fell to 94% ± 5% of the original viscosity. After 5 h the viscosity had decreased to 86% ± 8% of the original.

The observed decrease in viscosity supports a degradation of the polymer due to the solvent. Because of this degradation, all polymer solutions were used as close to the end of mixing as possible, where the viscosity remains constant. The unused polymer solution was discarded two hours after initial mixing to avoid large effects of polymer degradation.

### 3.2. Polymer Entanglement

The number of entanglements per polymer, *n_e_*, was calculated using the simple polymer volume theory described in the introduction section. We assumed the 3:1 ratio of acetic acid to formic acid behaved as a good solvent for PCL, and that the rapid evaporation of the solvent freezes the polymer in an entanglement state that is dependent upon, but not equivalent to, that of the entanglement of the initial polymer solution. Using this model, the onset of entanglement, when $n_{e}$ exceeds one, occurs at 4 g/dL PCL concentration for PCL with a number average molecular weight of 80 kg/mol. The number of entanglements per polymer, calculated for various PCL concentrations used in this study, can be found in Table 1.

**Figure 3:**
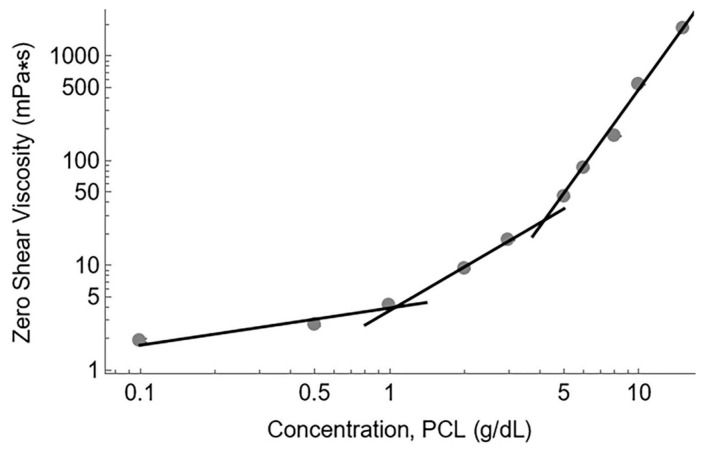
A log-log plot of zero-shear viscosity versus concentration. Gray circles represent the measured viscosity values. Standard deviation of each viscosity measurement is small, and error bars cannot be seen beyond the size of the data points. The intersection of the fit lines represents c* (1 g/dL) and c_e_ (4.5 g/dL), respectively.

To further support our calculations, we analyzed the zero-shear viscosity data versus the polymer concentration (Figure 3). From these data, the dilute, semi-dilute unentangled, and semi-dilute entangled regimes were identified. The intersection of linear fits for the dilute/semi-dilute unentangled and semi-dilute unentangled/semi-dilute entangled regimes provided values for c*, the critical concentration overlap, and c_e_, the entanglement concentration, respectively. The critical concentration overlap was approximately 1 g/dL PCL while the entanglement concentration was 4.5 g/dL PCL, in good agreement with the calculated onset of entanglement at 4 g/dL.

### 3.3. Characterization of Electrospun Fibers

**Figure 4:**
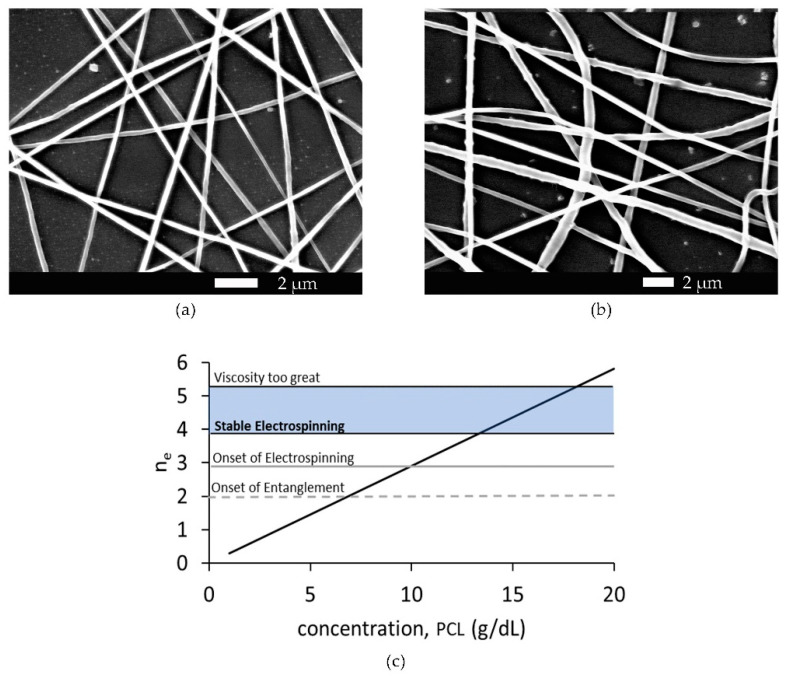
(**a**) SEM image of electrospun fibers formed from 13 g/dL PCL. (**b**) SEM image of electrospun fibers formed from 17 g/dL PCL. (**c**) The graph shows the number of entanglements, *n_e_*, as a function of weight/volume percent concentration of 80,000 M_n_ PCL. The dashed line crosses the function at the onset of entanglement at approximately 7 g/dL PCL. The solid gray line crosses the function at the onset of electrospinning at approximately 10 g/dl PCL, and the blue box indicates the region of stable electrospinning where smooth fibers were easily produced.

Electrospinning of the PCL solution was first achieved at a concentration of 10 g/dL PCL. At concentrations below 13 g/dL, PCL noncontinuous fiber and/or beaded fibers formed. At concentrations between 13 g/dL and 18 g/dL, PCL smooth continuous fibers formed during electrospinning. At and above 18 g/dL, the solution became too viscous for consistent electrospun fiber production, and the viscosity regularly impeded the solution from leaving the needle. Figure 4 displays the SEM images of 13 g/dL and 17 g/dL PCL concentrations, as well as a graph showing the relationship between polymer concentration, electrospun fiber production, and the number of polymer entanglements.

**Figure 5:**
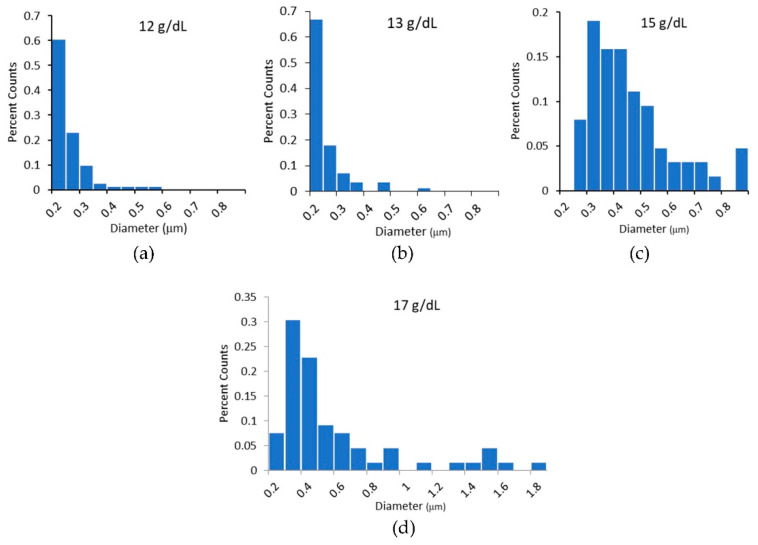
(**a**–**d**) Histograms of the diameters of electrospun fibers. In histogram (**c**), the 15 g/dL PCL histogram, all fibers above 800 nm are grouped together in one bin. In (**d**), 17 g/dL PCL fibers with diameters about 800 nm are not grouped together and no trend in diameter is evident at these large diameters.

We measured the diameters of the electrospun fibers using SEM. The diameters were measured from a minimum of five independently prepared samples and a minimum of 60 fibers at each polymer concentration. A Shapiro–Wilks test for normality indicated all sample populations were unlikely to come from a normal distribution. Diameter distributions had peaks in the range of 200 nm to 300 nm, with a long tail at higher diameters that approached zero between 600 and 800 nm. As polymer concentration increased, we measured a scattering of fibers with diameters above 800 nm (see Figure 5d). These larger diameter fibers, while few, have a large effect on the mean diameter of the distribution. Therefore, the average fiber diameters are reported with and without the inclusion of fibers greater than 800 nm in Table 2.

Many have reported a similar diameter dependence on concentration [52,53,54]. At lower viscosities, electrospun fibers formed under constant spinning parameters, such as collector distance and voltage, form smaller diameter fibers. The higher-viscosity polymer solutions offer greater resistance to the flow stresses and whipping stresses during electrospinning and form larger fibers.

### 3.4. Three-Point Bending Modulus

**Figure 6:**
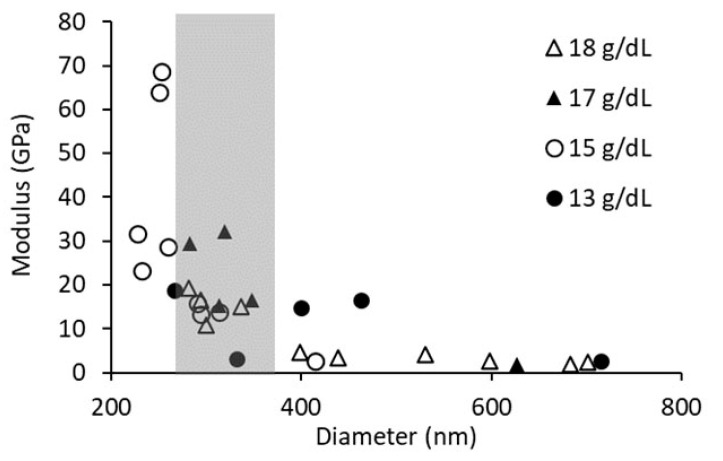
Graph of fiber diameter vs. three-point bending modulus. The gray area indicates a diameter range between 260 nm and 360 nm, which was used for modulus analysis.

The average fiber modulus was determined by three-point bending analysis for PCL concentrations of 13 g/dL, 15 g/dL, and 17 g/dL. Analyzing all fiber data, we see an expected modulus dependence on diameter (Figure 6). Therefore, fibers of similar diameter were selected to compare the moduli of different PCL solution concentrations, in an effort to minimize any effect attributable to diameter differences. The range of fiber diameters analyzed was between 260 nm and 360 nm. The results from this analysis are summarized in Table 2. We found no difference in modulus between the various PCL concentrations when equivalent diameters were analyzed.

### 3.5. Lateral Force Mechanical Properties

**Figure 7:**
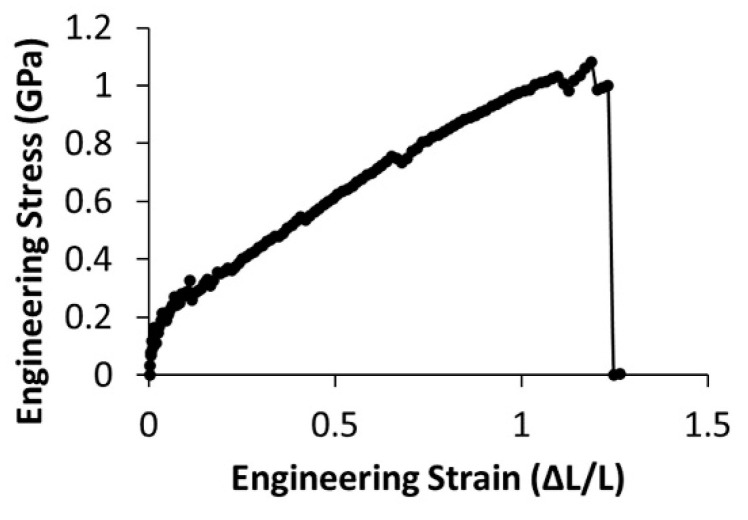
Representative engineering stress-strain curve for a fiber formed from 13 g/dL PCL, displaying strain softening.

Next, we tested the mechanical properties of the fibers using lateral manipulation via atomic force microscopy. We measured maximum extensibility, maximum stress, and modulus at 10% and 25% strain for PCL concentrations of 13 g/dL and 17 g/dL. We tested fibers with diameters ranging from 95 nm to 268 nm. All lateral force data were collected from at least three independent samples electrospun from separate polymer solutions. The stress-strain curves showed immediate strain softening (Figure 7).

Uncertainty in the position of the fiber, which is determined by force data and confirmed with optical microscopy, leads to large uncertainties in stress when the strain is small. For example, at 1% strain, the error in the fiber position propagates to a 15% uncertainty in stress, which reduces to a 5% uncertainty at 10% strain. Therefore, it is difficult to obtain accurate moduli at small strains, so the moduli are reported at 10% and 25% strain. The lower moduli measured at 25% strain are due to strain softening.

We found no significant difference in the average modulus between the PCL concentrations, consistent with the three-point bending data (*n* = 16, *p* > 0.05). As expected, and similar to Figure 5, we observed an increase in modulus as diameter decreased (not shown).

**Figure 8:**
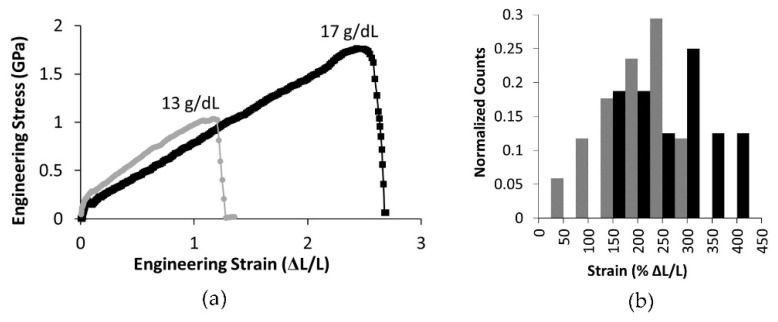
(**a**) Representative engineering stress-strain curves for 13 g/dL and 17 g/dL PCL. (**b**) A histogram of fiber extensibilities, with black bars showing data for 17 g/dL PCL and gray bars showing extensibilities for 13 g/dL.

Lateral atomic force microscopy mechanical tests, which probe mechanical properties at higher strains, showed significant differences in the mechanical properties of the two fiber populations. Figure 8 shows representative engineering stress versus strain curves for fibers formed from 13 g/dL and 17 g/dL PCL. As seen in the representative curves, the maximum strain and maximum stress of fibers formed from a 17 g/dL PCL concentration were significantly higher than those of the 13 g/dL PCL fibers (Table 3, *p* < 0.05 and *n* ≥ 16). Figure 8b displays a histogram of the extensibility data for 13 g/dL and 17 g/dL.

**Figure 9:**
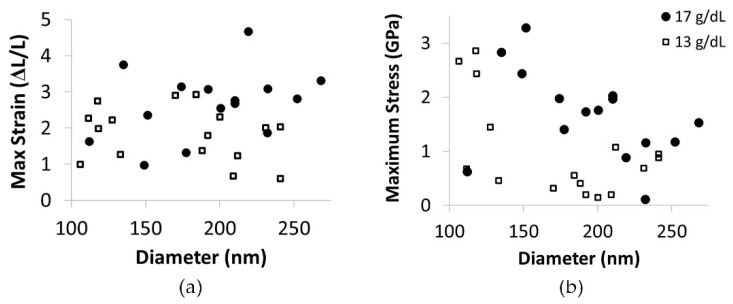
(**a**) A graph of maximum strain versus fiber diameter. Open squares represent data for 13 g/dL, and black circles are data for 17 g/dL PCL. Fiber extensibility does not display a dependence on fiber diameter. (**b**) A plot of maximum stress versus fiber diameter. Open squares represent data for 13 g/dL, and black circles are data for 17 g/dL PCL. The trend of increased modulus with decreased diameter is evident at each concentration. Additionally, the 17 g/dL fibers typically have a higher maximum stress than the 13 g/dL fibers at any given diameter.

The maximum strain did not depend on diameter in the range of diameters tested, Figure 9a. However, consistent with the modulus dependence on diameter, the maximum stress did increase as fiber diameter decreased (Figure 9b). Additionally, at all diameters, the 17 g/dL fibers typically had a higher maximum stress than the 13 g/dL fibers.

## 4. Discussion

Electrospinning is a complex process. The production of smooth continuous fibers using this method is dependent on solution properties, processing conditions and ambient conditions. In this study, we altered solution properties by increasing and monitoring the solution entanglement of PCL polymers. PCL is a biodegradable, biocompatible, and bioabsorbable polymer that shows promise for uses in biomedical and bioengineering applications. However, many solvents used for PCL electrospinning, such as dimethylformamide (DMF) and chloroform, are toxic [55,56,57]. Traces of these solvents left behind during the electrospinning process, for example, from incomplete solvent evaporation, could lead to issues for bio-related applications. Therefore, we chose to use 3:1 acetic to formic acid as a solvent due to its lower toxicity when compared to other common PCL solvents, thereby making our findings relevant for biological and other low-toxicity applications.

The use of the lower-toxicity solvents is not without their complications. Kanani et al. showed that the use of glacial acetic acid as a solvent for electrospinning PCL produced fibers with a nonuniform distribution of diameters, while the use of formic acid as a solvent produced uniform diameter populations [58]. In agreement with others, we found the combination of 3:1 acetic to formic acid produces a mostly uniform distribution of diameters, with peak diameters ranging between 200 and 300 nm under our processing conditions [59]. We did see a small number of higher-diameter fibers, especially as the concentration of PCL in the solution increased (Figure 5). Additionally, these solvents caused hydrolytic degradation of PCL, restricting the time that could elapse between polymer mixing and fiber production. Viscosity measurements indicated moderate degradation 5 h after the addition of the solvent to the polymer, Figure 2. Therefore, all fibers were produced within 5 h of the onset of mixing. However, longer times may be reasonable, but further investigation is needed.

Polymer entanglement has been identified as a critical element in solution spinnability, with electrospinning of smooth continuous fibers requiring polymer concentrations above the entanglement concentration [39,41,60,61]. Our results were consistent with these findings, and we found that the onset of stable electrospinning of non-beaded PCL, dissolved in 3:1 acetic to formic acid, occurred when the number of entanglements per polymer exceeded 3.8 (Figure 4). We were able to produce smooth continuous electrospun fibers of PCL dissolved in 3:1 acetic to formic acid solvent when the number of entanglements per polymer was between 3.8 and 5.2, corresponding to 80 kDa Mn PCL concentrations between 13 g/dL and 17 g/dL (Table 1).

The larger goal of this work was to identify the effect of polymer solution entanglement on the mechanical properties of the resultant electrospun fibers. Many groups have studied the mechanical properties of electrospun mats and individual fibers to better understand the origin of these properties and to better engineer electrospun fibers for their applications [62]. As polymer concentration increases in the solution, the number of polymer entanglements increases. The solution properties are affected by the electrospinning process. In particular, the flow stress as the solution is pushed through the needle, and then the stress due to jet extension and whipping caused by the interaction between the charge of the solution and the electric field lead to changes in the elongation and extension of the polymers. In an effort to minimize many of these effects, we held the flow rate through the needle and the electric field (volage and collector distance) constant for all experiments. In this manner, all entangled solutions were put through the same stress fields before fiber collection for analysis. The resultant polymer entanglement and supramolecular structure in the dried fiber will differ from that of the initial solution; however, they should depend upon their initial conditions. For example, solutions with the highest entanglement before electrospinning would be expected to produce fibers with the highest entanglement after drying. Therefore, we formed fibers from solutions with various numbers of entanglements and measured the mechanical properties of the resultant fibers (Table 2 and Table 3).

It is well documented that there is a strong relationship between fiber diameter and fiber modulus, and we see this relationship in our data (Figure 6) [20,43,45,63]. This modulus dependence on diameter leads to a steep growth in modulus over a small range of diameters and, therefore, can easily overwhelm other factors effecting fiber modulus. To reduce the effect of diameter on modulus, we analyzed fibers within a small range of diameters. To our surprise, we did not see an effect of the number of polymer entanglements on the modulus measured by either three-point bending or lateral force microscopy (Table 2 and Table 3). It should be noted that the diameter range for the three-point bending data was between 260 and 360 nm, while the diameter range for the lateral force data was from 95 to 268. In addition, the moduli determined by lateral force were acquired at a higher strain in a material that softened with strain. Therefore, the reported moduli differ for the two techniques due to the difference in sample diameter and strain.

Three-point bending tests measure mechanical properties using extremely small deformations, thereby causing small strains in the fibers. These small strains may not emphasize the influence of entanglement on fiber mechanical properties. Consider that the initial mechanism for fiber extension is for the polymers, whose shape can be simplistically visualized by a random walk, to align in the direction of the applied force and straighten. It should be noted that some inherent polymer alignment and straightening are expected due to the stresses inherent to the electrospinning process. However, low strain measurements may only probe further alignment and straightening. On the other hand, lateral manipulation by atomic force microscopy can probe fiber mechanics at moderate and large strains. As strain continues to increase, polymers may extend to the limit of their entangled confinement or slide past one another if polymer interactions such as entanglements or bonds do not restrict their movement.

The stress-strain curves measured using lateral force AFM displayed an immediate modulus decrease as strain increased, also referred to as strain softening (see Figure 7). Therefore, the modulus depended upon both fiber diameter and the strain at which the modulus was determined. The data for the modulus at 10% strain was significantly larger than the modulus measure at 25% strain (*p* < 0.01, Table 3). When comparing mechanical properties of electrospun fibers formed from polymer solutions with fewer entanglements per polymer, *n_e_*, to those with more entanglements per polymer, we found significant differences. Increasing *n_e_* from 3.8 to 4.9 correlated with an increase in extensibility by an average of 35% (Figure 8 and Table 3). The additional entanglements in the 17 g/dL PCL fibers may prevent the constituent polymers from slipping past one another and causing fiber failure. Due to their increased extensibility, fibers formed form solutions with greater polymer entanglement had a higher strength, or maximum stress before rupture (Figure 8). The average strength of the fibers increased by 65% in the range of polymer entanglement tested (Table 3). This behavior of increased strength with increased entanglement is consistent with work on polymer films [64].

Interestingly, fiber extensibility did not show a dependence on fiber diameter in the range of diameters tested; however, fiber strength did depend on diameter (Figure 9). This suggests the mechanisms responsible for increasing fiber modulus do not alter fiber extensibility, although both extensibility and modulus are often associated with changes in internal structure.

To the best of our knowledge, this is the first paper to report a change in individual fiber mechanics due to polymer solution entanglement changes. Our data suggest that polymer solution entanglement is an important parameter for tailoring individual electrospun fiber extensibility and strength. More work is needed to determine the robustness of this finding by altering entanglements using polymer molecular weight, changing the polymer solvent, testing for residual solvent, and investigating the relationship using different polymers.

Recent work by Alharbi et al. measured the extensibility of electrospun PCL fibers formed from PCL with different molecular weights [43]. However, the PCL concentration varied alongside the molecular weight, as this work was not focused on the effect of the *n_e_* on mechanical properties. Therefore, only a limited range of *n_e_* was studied, and under these conditions, no significant differences in extensibility were found. So, the question remains if changing molecular weight, and thereby changing *n_e_*, would produce similar outcomes. Other work on electrospun fiber mats has shown increased strength, initial modulus, and extensibility with increased polymer concentration [65]. However, many elements can contribute to altered mechanical properties of mats, such as the fiber morphology, fiber diameter distribution, fiber-fiber junctions, fiber density, and mesh porosity, as well as individual fiber mechanics.

The effect of electrospinning parameters on the ultimate mechanics and morphology of electrospun fibers is complex. The forces applied to the polymer solution between syringe and collector depend on collector distance, applied voltage, flow rate, and solution properties like conductivity and surface tension. In keeping the processing and ambient conditions constant, we found that for PCL dissolved in 3:1 acetic acid to formic acid, increasing the polymer concentration and, therefore, the polymer entanglement number leads to an increase in electrospun fiber extensibility and strength.

## 5. Conclusions

Reports of single electrospun fiber strength and extensibility are limited due to the difficulties in studying individual fibers of this size. Here we report for the first time the strength and extensibility of fibers formed from an 80,000 Mn PCL in a 3:1 acetic acid to formic acid solvent. More importantly, we investigate the mechanical properties of these fibers formed from polymer solutions with various polymer entanglement numbers. We adjusted the polymer solution concentration to change the polymer entanglement. We show, for the first time to our knowledge, an increase in single electrospun fiber strength and extensibility as polymer concentration increases and attribute these changes to polymer entanglement. Understanding single fiber properties and their underlying mechanisms is important for predicting and tailoring the mechanical properties of large-scale structures comprising electrospun fibers. While further work is needed to ensure this relationship between entanglement, strength, and extensibility is robust for other polymers, this work provides initial support for the expectation that polymer entanglement via polymer concentration enhances individual electrospun fiber mechanics.

## Figures and Tables

**Table 1:** Properties of the polymer solution and resultant electrospun fibers. Values are the average ± the standard deviation where applicable.

PCL (g/dL)	*n_e_*	Electrospinning Ability	Diameter (nm)	Diameter (excluding >800 nm)	Percent of Fibers >800 nm
12	3.5	Beaded and Smooth fibers	210 ± 70	210 ± 70	0%
13	3.8	Smooth fibers	200 ± 80	200 ± 80	0%
15	4.4	Smooth fibers	410 ± 170	390 ± 120	5%
17	4.9	Smooth fibers	550 ± 390	390 ± 130	18%

**Table 2:** Analysis of fibers with diameters between 260 nm and 360 nm. Values are the average ± the standard deviation.

Concentration (g/dL)	Avg Diameter AFM (nm)	Three-Point Bending Modulus (GPa)
13	300 ± 50	10.9 ± 11.1
15	290 ± 20	17.8 ± 7.3
17	320 ± 30	23.3 ± 8.7
18	300 ± 20	15.4 ± 3.5

**Table 3:** Lateral Force AFM data.

Concentration (g/dL)	Diameter (nm)	Max Strain (%)	Max Stress (GPa)	10% Modulus (GPa)	25% Modulus (GPa)
13	174 ± 48	180 ± 70	1.0 ± 0.9	2.9 ± 3.2	1.7 ± 2.0
17	188 ± 50	260 ± 100	1.7 ± 0.8	3.2 ± 2.6	2.0 ± 1.7
		*p* < 0.05	*p* < 0.05		

## Data Availability

Analyzed data are contained within the article. The detailed data presented in this study are available on request from the corresponding author.
